# COL10A1 promotes tumorigenesis by modulating CD276 in pancreatic adenocarcinoma

**DOI:** 10.1186/s12876-023-03045-2

**Published:** 2023-11-16

**Authors:** Qiaodong Xu, Jieting Zheng, Zegeng Su, Binlie Chen, Songgang Gu

**Affiliations:** 1 Department of Hepatobiliary surgery, Cancer Hospital of Shantou University Medical College, No. 7 Raoping Road, Shantou, 515041 China; 2 Department of pharmacy, Cancer Hospital of Shantou University Medical College, No. 7 Raoping Road, Shantou, 515041 China; 3 Department of anesthesiology, Cancer Hospital of Shantou University Medical College, No. 7 Raoping Road, Shantou, 515041 China

**Keywords:** Pancreatic adenocarcinoma, COL10A1, Bioinformatics analysis, CD276

## Abstract

**Background:**

Pancreatic adenocarcinoma (PAAD) is a lethal malignant tumour. Further study is needed to determine the molecular mechanism and identify novel biomarkers of PAAD.

**Methods:**

Gene expression data from the GSE62165 microarray were analysed with the online software Morpheus to identify differentially expressed genes (DEGs). The STRING database was used to generate a protein‒protein interaction (PPI) network for these DEGs. Hub genes were identified with Cytoscape. COL10A1 expression in PAAD was analysed via the GEPIA database. COL10A1 expression in pancreatic cancer cell lines was measured by using qRT‒PCR. The LinkedOmics database was utilized to perform survival analysis of pancreatic adenocarcinoma patients grouped based on COL10A1 expression level. CCK-8, wound healing, and Transwell assays were used to study the role of COL10A1 in pancreatic cancer cell viability, migration, and invasion. Differentially expressed genes that were related to COL10A1 in PAAD were analysed via the LinkedOmics portal. After COL10A1 was knocked down, CD276 expression was assessed by western blotting.

**Results:**

COL10A1 was identified as one of the hub genes in PAAD by bioinformatics analysis of the GSE62165 microarray with Morpheus, the STRING database and Cytoscape. GEPIA revealed elevated expression of COL10A1 in PAAD samples vs. normal samples. COL10A1 expression was also increased in pancreatic cancer cells vs. control cells. Survival analysis of PAAD patients via LinkedOmics revealed that high expression of COL10A1 was associated with a poorer prognosis. Knockdown of COL10A1 inhibited the proliferation, migration, and invasion of cells in functional assays. Furthermore, mechanistic studies indicated that CD276 was a target of COL10A1 and that knockdown of COL10A1 decreased CD276 expression. Overexpression of CD276 in cells reversed COL10A1 knockdown-induced repression of proliferation and migration.

**Conclusions:**

Our research suggests that COL10A1 promotes pancreatic adenocarcinoma tumorigenesis by regulating CD276. This study provides new insight into biomarkers and possible targets for pancreatic cancer treatment.

**Supplementary Information:**

The online version contains supplementary material available at 10.1186/s12876-023-03045-2.

## Introduction

According to GLOBOCAN data, there were 495,773 new cases of pancreatic cancer in 2020, accounting for 2.6% of all cancer cases. Although pancreatic cancer is the 14th most prevalent cancer, it ranks 7th in terms of cancer-related deaths [1]. The main causes of pancreatic cancer include genetic factors, smoking, high-fat diet consumption, obesity, diabetes, chronic pancreatitis and nitrite intake [2, 3]. The most common type of pancreatic cancer is pancreatic adenocarcinoma (PAAD). The features of PAAD include early metastasis, high aggressiveness, and insensitivity to chemotherapy or radiotherapy. Only 20% of patients with localized cancer qualify for curative surgical resection [4]. Because of the lack of an effective predictive index, most patients are diagnosed with PAAD when their tumours have infiltrated surrounding tissues or metastasized to distant sites. The median overall survival of patients in the advanced stage who received systemic chemotherapy instead of radical surgical treatment is approximately 8.5 months [5]. Carbohydrate antigen 199 (CA-199) is the most commonly used biomarker in the diagnosis of PAAD. However, the sensitivity of CA-199 in diagnosing pancreatic cancer is only approximately 52.7% [6]. To improve the outcome of PAAD patients, additional molecular markers or therapeutic targets must be identified. Recently, high-throughput sequencing technology has been widely used to identify genes that are differentially expressed during tumorigenesis, and this approach is used to seek potential diagnostic and therapeutic targets [7]. Bioinformatics analysis of tumour-related microarrays may provide novel insights into the molecular mechanism underlying pancreatic oncogenesis.

Collagen type X alpha 1 (COL10A1) is a member of the collagen family. Collagens play important roles in tissue organization and structure. They are important regulators of cell proliferation, migration, and differentiation and interact with cells via some receptor families [8]. Previous studies have shown that COL10A1 expression is upregulated in multiple solid cancers, including breast, lung, oesophageal, stomach, colon, and bladder cancers [9, 10]. Furthermore, COL10A1 expression was found to be abnormally elevated in colorectal cancer vs. normal controls and inversely correlated with the outcome of colorectal cancer patients [11]. COL10A1 also promotes the malignant progression of gastric cancer and may be a therapeutic target and predictive biomarker in GC patients [12]. Several studies have revealed that collagen, as one of the major components of the tumour microenvironment, is closely associated with the progression of pancreatic cancer. Deletion of type I collagen in myofibroblasts was reported to contribute to immune suppression and malignant progression in pancreatic cancer [13]. COL11A1 is a potential biomarker of pancreatic cancer [14]. Pancreatic stellate cell-derived COL1A1 promotes the migration and invasion of pancreatic cancer cells [15]. However, the role and function of COL10A1 in PDAC have not been elucidated.

CD276, also known as B7-H3, belongs to the immune modulatory factor family that includes PD-L1 (B7-H1), which is involved in the regulation of the immune response to tumour cells [16]. CD276 has been identified as a promising and attractive target for immunotherapy of cancers due to its abnormal upregulation in many types of tumours and participation in tumour progression [17]. Currently, immunotherapy with PD-1/PD-L1 inhibitors is effective for treating malignant melanoma, non-small cell lung cancer and other tumours but not for treating pancreatic cancer. Recent studies have revealed that CD276 is significantly upregulated in pancreatic cancer, and high CD276 expression in patients is associated with a poor prognosis [18]. Another study found that the combination of CD276 inhibitors and PD-1 inhibitors was a promising treatment for non-small cell lung cancer that did not react to PD-1 inhibitors but expressed CD276 [19]. This serves as a reminder that CD276 inhibitors alone or combined with PD-1 inhibitors may be a new direction for pancreatic cancer therapy.

According to our study, we first analysed the data of the GSE62165 microarray, which contained the gene expression information of 118 pancreatic ductal adenocarcinoma samples and 13 control (nontumour) pancreatic samples, with the online software Morpheus. Based on a *P* value of 0.05 and |log2(fold change)|>1, 68 DEGs between normal pancreatic tissue tumours and PDAC were identified. Then, 10 hub genes, including COL10A1, were identified employing Cytoscape based on the PPI network of the DEGs that was generated using the online database STRING. COL10A1 was downregulated in normal pancreatic tissues compared with PDAC tissues in the bioinformatics analysis of GSE62165. We further explored the role of COL10A1 in pancreatic adenocarcinoma. Significant overexpression of COL10A1 was observed in pancreatic adenocarcinoma tissues vs. normal pancreatic tissues according to GEPIA. In addition, a significant increase in COL10A1 expression was observed in pancreatic cancer cells. Survival analyses using the LinkedOmics database revealed that high COL10A1 expression was associated with a worse outcome in PAAD patients. Moreover, COL10A1 knockdown repressed cell viability, invasion, and migration in vitro. The differentially expressed genes related to COL10A1 in PAAD were analysed via the LinkedOmics portal. COL10A1 was positively associated with CD276, which is an immune checkpoint molecule that plays a vital role in regulating the malignant cellular-biological behaviour of tumours. In addition, knockdown of COL10A1 repressed CD276 expression. Overexpression of CD276 reversed COL10A1 knockdown-induced repression of proliferation and migration. Collectively, the results of our study indicate that COL10A1 facilitates tumour progression by upregulating CD276 in pancreatic cancer.

## Materials and methods

### Bioinformatics analysis

The GEO database (http://www.ncbi.nlm.nih.gov/geo/) provided detailed gene expression information for the GSE62165 microarray. Differentially expressed genes were identified using the online tool Morpheus (https://software.broadinstitute.org/morpheus/) (fold change = normal pancreatic tissue gene expression/PDAC tissue gene expression, *P* value < 0.05 and |log2(fold change)|>1). The downstream analysis was based on the genes with the largest fold changes in expression among those that were repeated. A heatmap of DEGs was subsequently generated. Using the online database STRING (https://string-db.org/), a PPI network of the identified DEGs was generated. In addition, 10 hub genes were identified using Cytoscape’s cytoHubba plug-in. The topological analysis method we selected in the cytoHubba plug-in was the maximal clique centrality (MCC) method. On the basis of TCGA and GTEx data, GEPIA (http://gepia.cancer-pku.cn/) was applied to analyse the gene expression of COL10A1 in pancreatic adenocarcinoma (PAAD) tissues and normal pancreatic tissues. Based on TCGA data, survival analysis of PAAD patients grouped based on COL10A1 expression level were conducted using the LinkedOmics database (http://www.linkedomics.org/login.php). Differences were considered significant at a P value of less than 0.05.

### Cell culture

The Cell Bank of Chinese Academy of Sciences (Shanghai, China) provided three human pancreatic cancer cell lines (Panc-1, BxPC-3, and ASPC-1 cells) as well as a pancreatic duct epithelial cell line (HPDE6-C7 cells). DMEM supplemented with 10% foetal bovine serum, 1% L-glutamine, 100 g/mL streptomycin, and 100 g/mL penicillin was used to culture Panc-1 and BxPC-3 cells. A mixture of 10% foetal bovine serum, 1% L-glutamine, 100 g/mL streptomycin, and 100 g/mL penicillin was added to RPMI 1640 medium (Gibco, CA, USA), and this medium was used to culture ASPC-1 and HPDE6-C7 cells. A 37 °C incubator (Thermo, USA) with 5% CO2 was used to maintain all the cell lines.

### RNA extraction and quantitative real-time PCR analysis

Total RNA was extracted from cells 36 hours after transfection using TRIzol reagent (Invitrogen, Carlsbad, USA). An RNA reverse transcription kit (Takara, Dalian, China) was used to convert total RNA to cDNA. qRT‒PCR analysis was performed by using Power SYBR Green (Takara, Dalian China). Quantification was performed using the 2−∆∆CT method, and the results are presented as fold changes. The expression of the gene of interest was normalized to that of β-actin. The primer sequences (forward and reverse, 5’-3’) were as follows: COL10A1, CCCTCTTGTTAGTGCCAACCA, GGCCTACCCAAACATGAGTCC; β-actin, CTACAGGGACGCCATCGAATC, AGCCCTCTTCAGCTTGTGTTG.

### Transfection of COL10A1-specific siRNAs into pancreatic cancer cells

Panc-1 and BxPC-3 cells were seeded in six-well plates in DMEM supplemented with 10% FBS. Using Lipofectamine 2000 (Invitrogen, Thermo Fisher Scientific), the cells were transfected overnight with specific siRNA against COL10A1 and scrambled control siRNA (Invitrogen, Thermo Fisher Scientific). After transfection, the mRNA expression of COL10A1 was evaluated by real-time RT‒PCR.

### Transient overexpression of CD276 in pancreatic cancer cells

The open reading frame of CD276 was cloned into the retroviral vector pMSCV-eGFP-Puro (Clontech, Mountain View, CA, USA) according to the manufacturer’s instructions. Panc-1 and BxPC-3 cells were transfected with the plasmid by using Lipofectamine reagent (Invitrogen, CA, USA).

### Transwell cell invasion assay

Cells were seeded in 6-well plates one day in advance. The siRNA oligos or controls were transferred into the cells by using Lipofectamine 2000, and the DMEM medium was changed 6 h later. The cells were then cultured for 48 h in 5% CO2. To conduct the Transwell experiment, 2 × 10^5^ Panc-1 cells in serum-free DMEM medium were added to precoated upper chambers of 8-μM pore Transwell plates that were coated with 1:8 Matrigel. A volume of 500 μL of DMEM supplemented with 20% FBS was added to the lower chambers. The culture plates were incubated in an incubator (37 °C, 5% CO_2_) for 72 h. Cells in the upper chamber were removed, and the cells that passed through to the lower chamber were fixed with 4% paraformaldehyde and stained with crystal violet. The staining results were observed under a microscope(OLYMPUS, Japan)and photographed. Cells were counted under a microscope at 100×, and 3 fields were randomly selected for cell observation and counting.

### CCK-8 assay

Cells were transfected with COL10A1 siRNA and scramble siRNA and incubated at 37 °C in a 5% CO_2_ incubator (Thermo, USA) in 96-well culture plates (5000 cells per well). The next day, the cells were washed twice with PBS, and then 100 μl DMEM was added to each well. The old solution was discarded after 6 h, 24 h, 48 and 72 h. Ten microlitres of CCK-8 and 90 μl of medium were placed in each well and incubated for 2 h each at 37 °C. Each group’s cell proliferation ability was calculated based on the OD values at 450 nm measured by a microplate reader (BioTek Instruments, Inc., Winooski, VT, USA).

### Wound healing assay

Six-well plates were coated with transfected cells (5 × 10^4^ cells per well) in DMEM medium. When the cells reached 90% confluence, a linear wound was created across the monolayer with a sterile pipette tip. The entire plate was rinsed with PBS three times to wash off the cell debris that was generated by the scratch. For 72 h, the cells were cultured in medium with low serum levels. The cells were observed and photographed at 0 and 24 h after scratching. By using a microscope(OLYMPUS, Japan), we observed the migration process after washing the cells twice with PBS. We measured the scratch width in 5 fields of view for each group of cells and calculated the migration distance using Image Pro-Plus 6.0 software. Cell migration ability was quantified based on wound width as follows: migration rate = (the scratch width at 0 h - the scratch width at other time points)/the scratch width at 0 h.

### Clone formation

The cells were digested with trypsin and then counted after being resuspended in complete culture medium (DMEM medium with 10% fetal bovine serum). 700 cells per well were inoculated in a 6-well culture plate. The culture was continued for 14 days, with the medium being changed every 3 days. After cloning, the cells were photographed under a microscope and then washed once with PBS. Next, 1 mL of 4% paraformaldehyde was added to each well for fixation for 30–60 min. Following fixation, 1 mL of crystal violet staining solution was added to each well to stain the cells for 10–20 min. The cells were rinsed several times with PBS, air-dried, and photos were taken using a digital camera (Nikon, Japan).

### Differentially expressed genes correlated with COL10A1 in PAAD

Analysis of the differentially expressed genes in PAAD that were correlated with COL10A1 was conducted via the LinkFinder module of LinkedOmics. A Spearman correlation test was used to statistically analyse the results. Volcano plots and heatmaps were used to present all the results graphically.

### Western blotting

RIPA buffer (Beyotime, Shanghai, China) was used to lyse and isolate proteins from transfected cells. BCA working reagent (Beyotime, Shanghai, China) was used to measure the absorbance of the protein samples at 562 nm after 30 min of incubation. The protein concentration of each sample was calculated using a standard curve. An SDS‒PAGE gel was used to separate protein samples, and then, the proteins were transferred to a PVDF membrane. After blocking, the PVDF membranes were incubated with anti-CD276 (1:1000; Proteintech, 14453-1-AP, China), anti-β-actin (1:1000; Boster, BM0627, China) and anti-GAPDH (1:1000; Proteintech, 10494-1-AP, China) at 4 °C overnight. HRP-conjugated secondary antibody (Goat Anti-rabbit IgG, Boster, BA1055, China) and (Goat Anti-mouse IgG, Boster, BA1051, China) were then incubated with the membranes for 1 h at 25 °C. An ECL detection system was used to detect the immunoreactive bands after the membrane was washed three times.

### Statistical analysis

This study was conducted using SPSS 19.0 and GraphPad Prism 8.0 for analysis, and the data are presented as the mean ± SD. To analyse differences among multiple groups, one-way analysis of variance (ANOVA) was performed, followed by Bonferroni post hoc tests. The difference between two groups was analysed using Student’s t test. A P value of less than 0.05 was considered to indicate statistical significance.

## Results

### COL10A1 is a hub gene in PDAC according to bioinformatics analysis of GSE62165

**Fig. 1:**
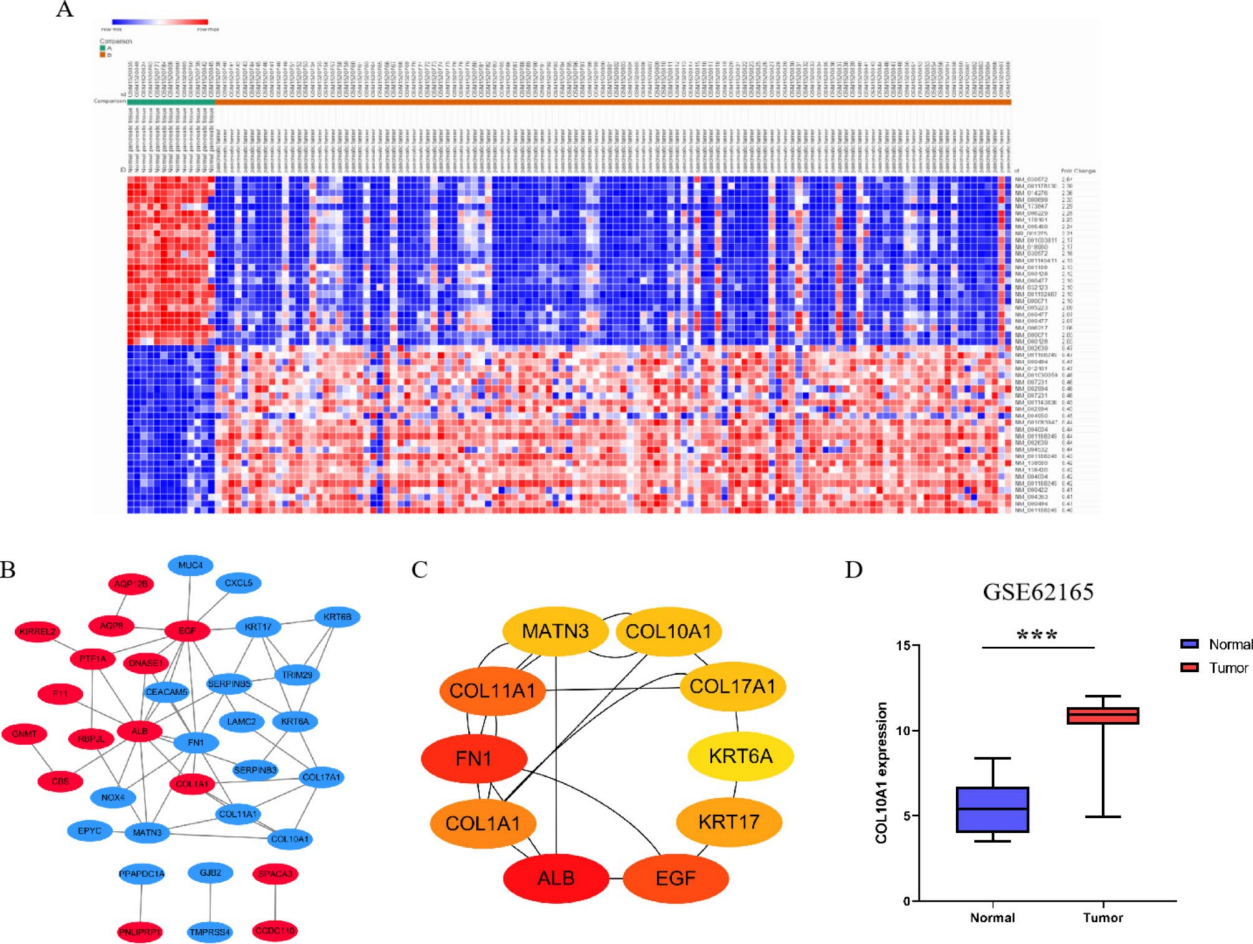
COL10A1 is a hub gene in PDAC according to bioinformatics analysis of the GSE62165 dataset. (**A**) Heatmap of the top 25 upregulated and top 25 downregulated DEGs. Red: upregulation; blue: downregulation. (**B**) PPI network of DEGs. The red nodes indicate upregulated genes, and the blue nodes indicate downregulated genes. (**C**) Top 10 hub genes identified by Cytoscape. (**D**) COL10A1 expression levels in normal pancreatic tissues versus PAAD tissues from the GSE62165 dataset

The gene expression data of the GSE62165 microarray were downloaded from the GEO database and analysed by the online tool Morpheus. *P* value ≤ 0.05 and |log2(fold change)|>1 were used to identify DEGs between 13 normal pancreatic tissues and 118 PDAC tissues. Forty-three downregulated genes and 25 upregulated genes were verified. A heatmap of the top 25 upregulated and downregulated DEGs (ranked according to the fold change, fold change = normal pancreatic tissue gene expression/PDAC tissue gene expression) was generated via Morpheus as a reference (Fig. 1A). After analysing the PPI network, we identified hub genes. The downstream analysis was based on genes with the largest fold changes in expression among those that were repeated. Twenty-one remaining upregulated and 26 remaining downregulated DEGs were then analysed by using the STRING database to produce a network diagram. Then, the PPI network of these DEGs was developed by using Cytoscape software (Fig. 1B). Furthermore, the top 10 hub genes (MATN3, COL10A1, COL17A1, KRT6A, KRT17, EGF, ALB, COL1A1, FN1, COL11A1) were identified by the cytoHubba plug-in in Cytoscape using the calculation method of maximal clique centrality (Fig. 1C). According to previous studies, most of the 10 identified hub genes have been studied in pancreatic cancer, with the exception of COL10A1. The expression of COL10A1 was lower in normal pancreatic tissues than in PDAC tissues in the bioinformatics analysis of GSE62165 (Fig. 1D). Many solid tumour types, including oesophageal cancer, lung cancer, breast cancer, stomach cancer, colon cancer, and bladder cancer et al., have been shown to have elevated levels of COL10A1. In addition, colorectal cancer patients with high COL10A1 expression presented a poorer prognosis than those with low expression. COL10A1 appears to promote the malignant behaviour of gastric cancer cells, making it a potential therapeutic target and biomarker of gastric cancer. However, the role of COL10A1 in pancreatic cancer remains unclear.

### COL10A1 expression is elevated in pancreatic adenocarcinoma and associated with a poor prognosis

**Fig. 2:**
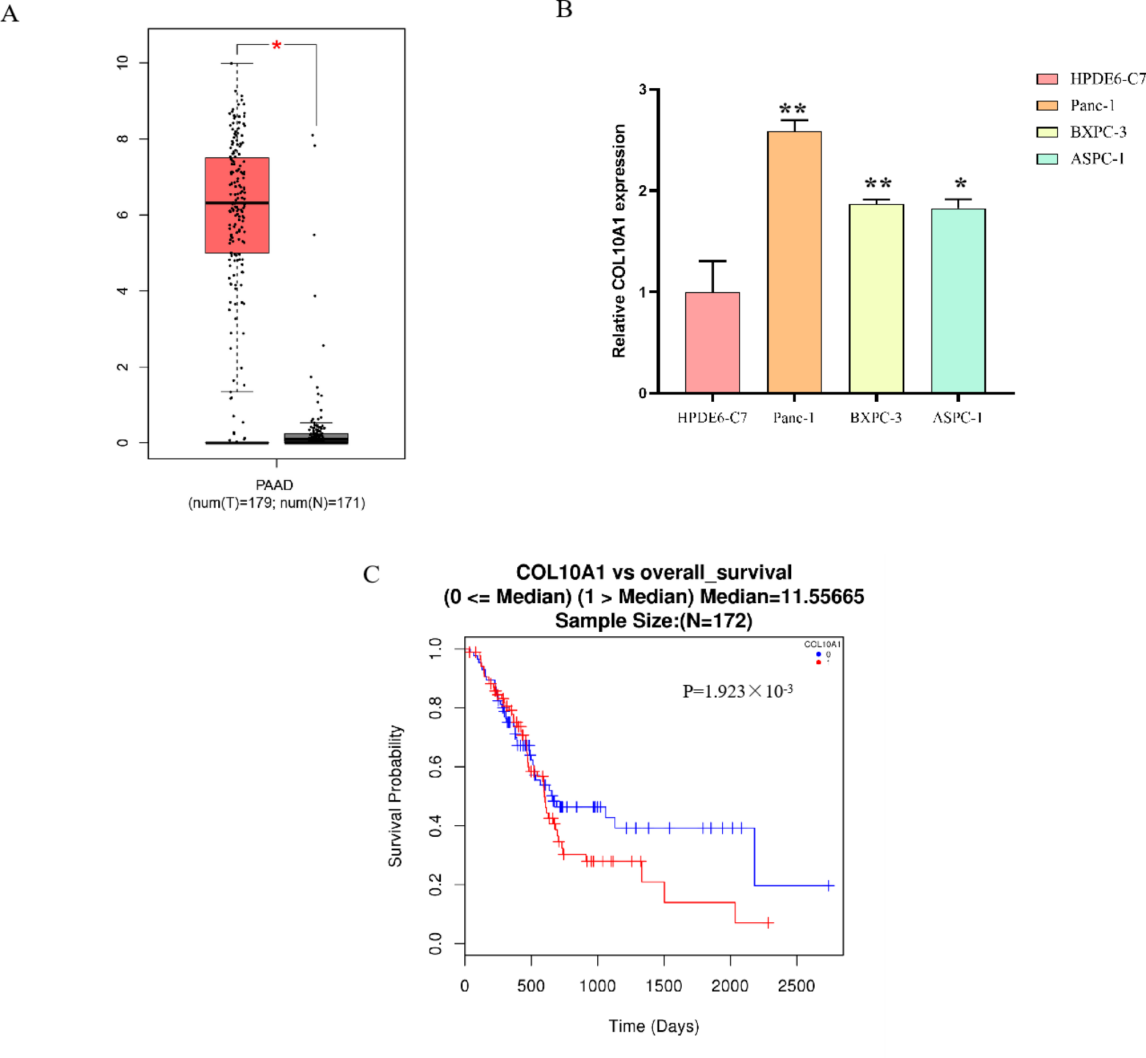
COL10A1 is elevated in pancreatic adenocarcinoma and associated with a poor prognosis in PAAD patients. (**A**) The mRNA expression of COL10A1 in pancreatic adenocarcinoma tissues (n = 179) and normal pancreatic tissues (n = 171) (*, P < 0.05). (**B**) COL10A1 expression in pancreatic cell lines (Panc-1, BxPC-3, ASPC-1 cells) and the human normal pancreatic duct epithelial cell line HPDE6-C7 was examined by qRT‒PCR (*, P<0.05; **, P<0.01). (**C**) Overall survival (OS) analysis of PAAD patients grouped based on COL10A1 expression level using the LinkedOmics database (P = 1.923 × 10^− 3^)

We tried to explore the role of COL10A1 in pancreatic cancer by analysing the data in a public database. By using the GEPIA database, we analysed COL10A1 mRNA expression in PAAD. Pancreatic adenocarcinoma tissues (n = 179) exhibited increased expression of COL10A1 compared to normal pancreatic tissues (n = 171) (P < 0.05, Fig. 2A). Moreover, COL10A1 was upregulated in the BxPC-3, Panc-1, and ASPC-1 pancreatic cancer cell lines compared with the human normal pancreatic duct epithelial cell line HPDE6-C7 (Fig. 2B). Overall survival (OS) analysis using the LinkedOmics database revealed that PAAD patients with high COL10A1 expression had shorter overall survival than those with low expression (P = 1.923 × 10^− 3^, Fig. 2C).

### Knockdown of COL10A1 inhibits the proliferation, invasion, and migration of Panc-1 pancreatic cancer cells

**Fig. 3:**
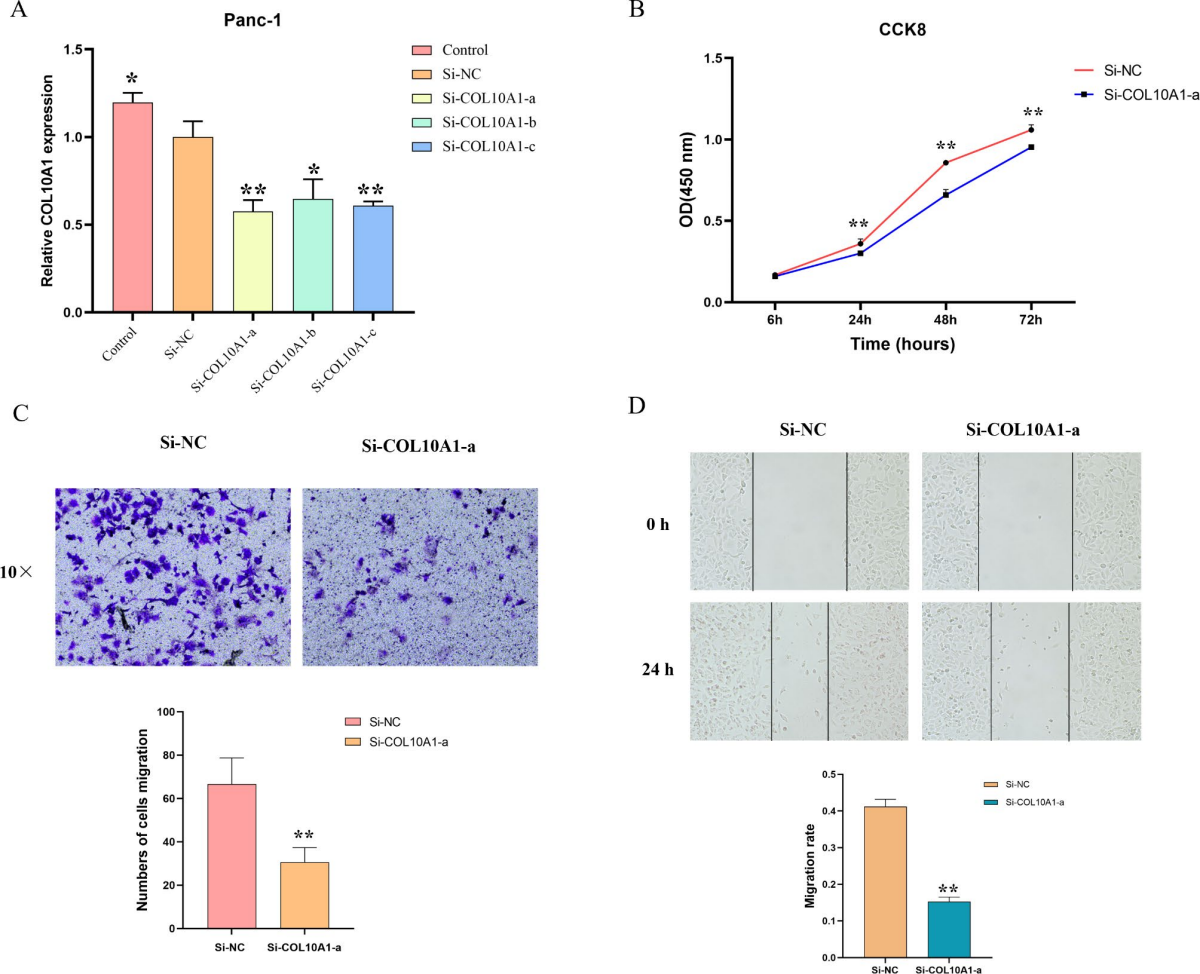
Knockdown of COL10A1 inhibits the proliferation, invasion, and migration of Panc-1 pancreatic cancer cells. (**A**) qRT‒PCR results of COL10A1 expression in different experimental groups of Pan-1 cells: the normal control, siRNA negative control (si-NC), si-COL10A1-a, si-COL10A1-b, and si-COL10A1-c groups (*, P<0.05; **, P<0.01). (**B**) CCK-8 assay results of the si-NC and si-COL10A1-a groups at 6 h, 24 h, 48 h, and 72 h time intervals. (*, P < 0.05; **, P < 0.01). (**C**) Transwell assay results showed that the si-COL10A1-a group had a weaker invasive ability. Microscopy images. (*, P < 0.05; **, P < 0.01). (**D**) Scratch wound healing assay results. Images of the si-NC and si-COL10A1-a groups at 0 and 24 h postinjury. Microscopy images. (*, P < 0.05; **, P < 0.01)

The knockdown efficiencies of three siRNAs targeting COL10A1 in Panc-1 cells with high COL10A1 expression were measured by qRT‒PCR and compared to the effect of a control siRNA. Si-COL10A1-a, which produced the most significant knockdown results, was chosen for subsequent studies (Fig. 3A). In CCK-8 assays, the OD value at 450 nm decreased significantly in the COL10A1 knockdown group compared with the control group (Fig. 3B). This indicates that knockdown of COL10A1 has a proliferation-inhibiting effect on Panc-1 cells. Panc-1 cell invasion and migration were significantly suppressed when COL10A1 expression was silenced according to Transwell assays and wound healing assays (Fig. 3C and D).

### COL10A1 regulates CD276 expression in pancreatic cancer cells

**Fig. 4:**
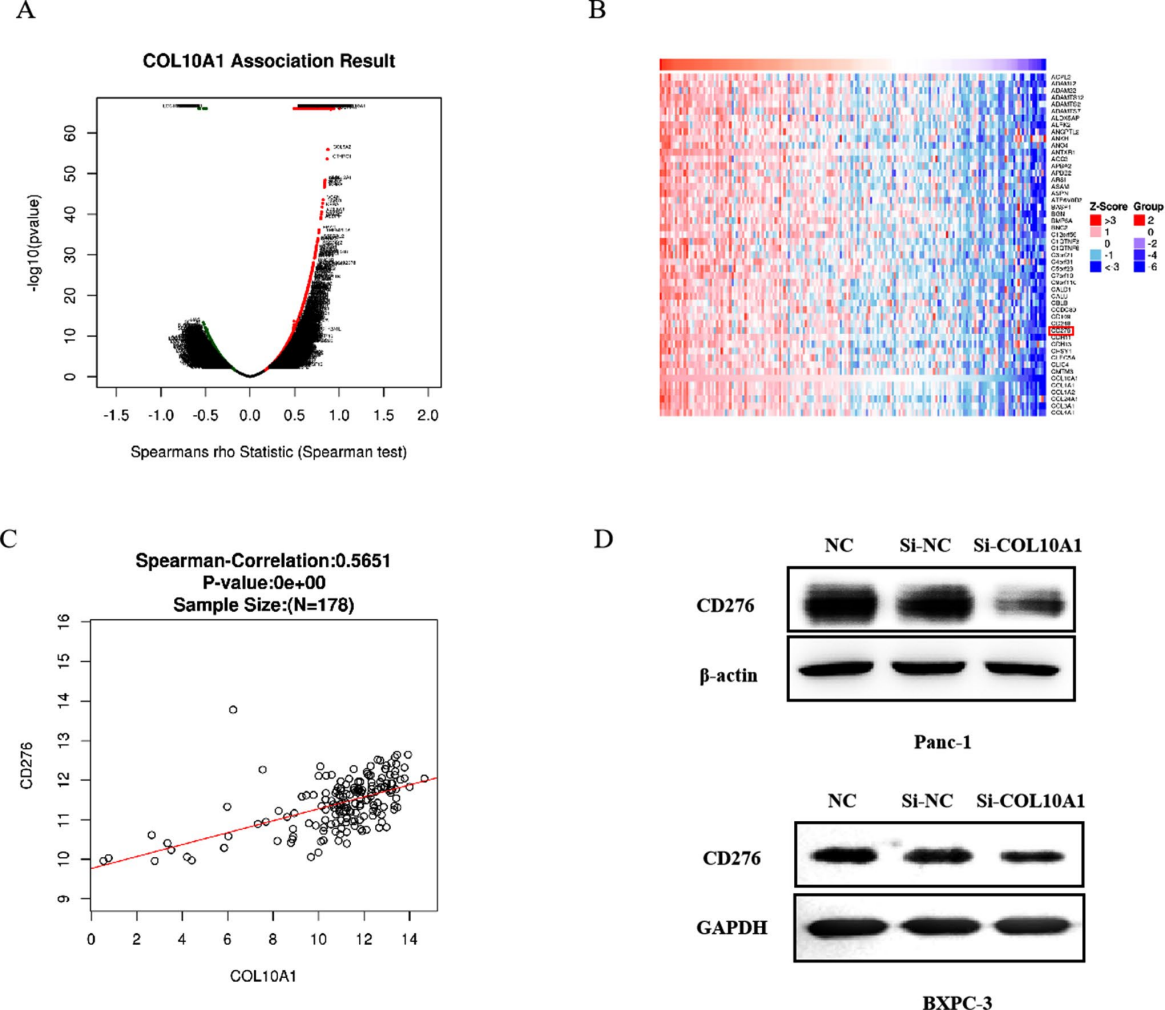
COL10A1 regulates CD276 expression in pancreatic cancer cells. (**A**) A Spearman test was used to analyse correlations between COL10A1 and differentially expressed genes in PAAD. (**B**) Heatmaps showing genes positively correlated with COL10A1 in PAAD (top 50). Red indicates positively correlated genes, and green indicates negatively correlated genes. (**C**) The correlations between COL10A1 and CD276 expression in PAAD were analysed with Spearman’s rank correlation method by LinkedOmics. (**D**) After knockdown of COL10A1, the expression of CD276 was assessed by western blotting in Panc-1 and BXPC-3 cells

Using the LinkFinder module in LinkedOmics, TCGA data for 178 patients with PAAD were assessed to identify differentially expressed genes related to COL10A1. The results are presented in a volcano plot (Fig. 4A). COL10A1 expression was positively correlated with the expression of 3170 genes (dark red dots) and negatively correlated with the expression of 2684 genes (dark green dots) (false discovery rate [FDR] < 0.01). The top 50 genes with significant positive correlations with COL10A1 are presented in the heatmap (Fig. 4B). According to the analysis results of LinkedOmics, CD276 expression was strongly positively associated with COL10A1 expression (Spearman correlation = 0.5651, Fig. 4C). CD276 suppresses the cytotoxic effect of T cells on tumours and is involved in the immune escape of tumour cells. Overexpression of CD276 has been observed in many solid tumours and inversely correlated with the prognosis of patients. Recent studies have revealed that high CD276 expression is independently correlated with poor pathological differentiation status, lymph node metastasis, and poor survival in pancreatic cancer patients. After knockdown of COL10A1, we evaluated CD276 expression using western blotting. According to the results, knockdown of COL10A1 repressed CD276 expression in pancreatic cancer cells (Fig. 4D).

### COL10A1 promotes PAAD progression through CD276

**Fig. 5:**
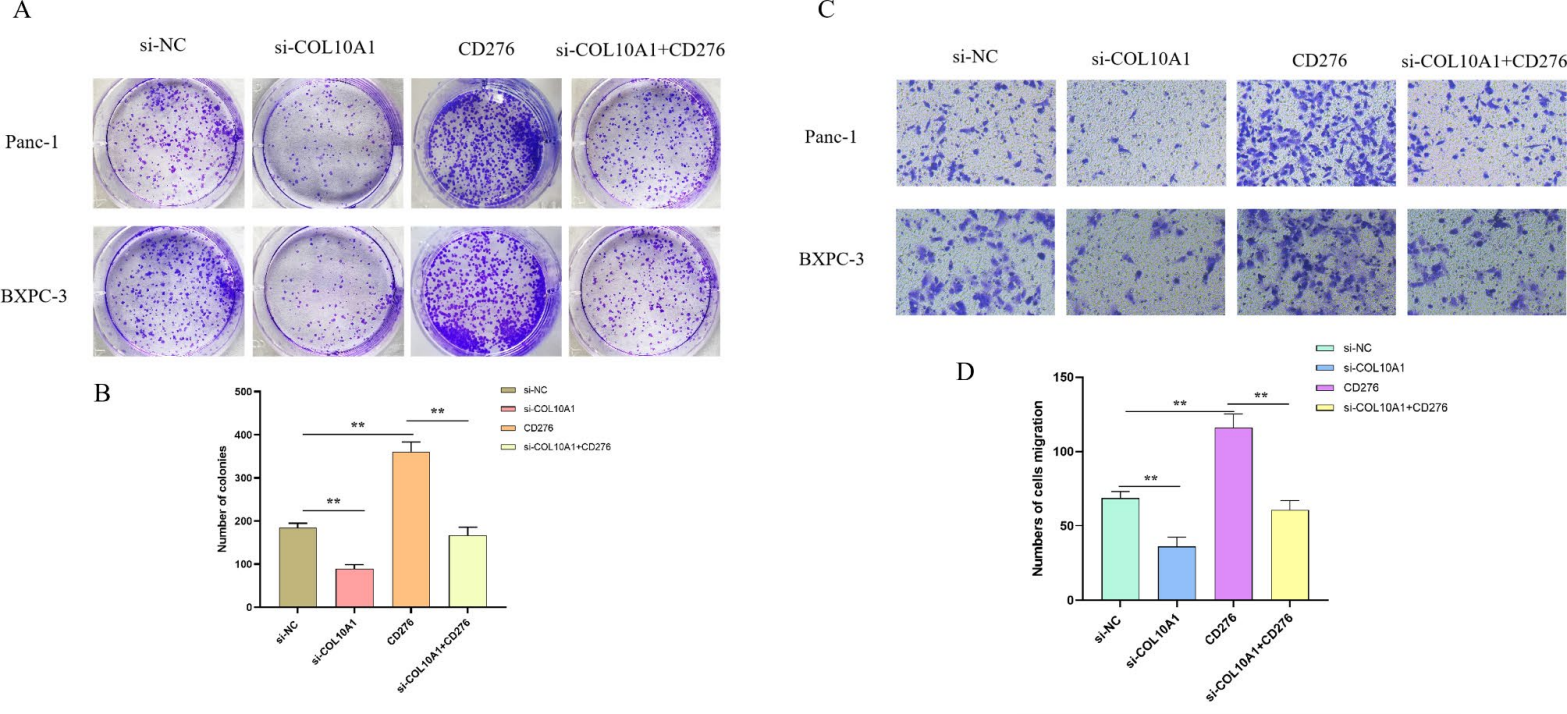
COL10A1 promotes PAAD progression through CD276. Colony formation (**A**, **B**) and Transwell (**C**, **D**) assays were performed to assess changes in cell proliferation and migration in response to knockdown of COL10A1 combined with overexpression of CD276 in Panc-1 and BXPC-3 cells

We performed colony formation and transwell assays to explore whether COL10A1 promotes PAAD progression by regulating CD276. COL10A1 was transiently knocked down, while CD276 was transiently overexpressed in pancreatic cancer cells. Knockdown of COL10A1 repressed the proliferation and migration of cells, but overexpression of CD276 in COL10A1 knockdown cells reversed this effect (Fig. 5).

## Discussion

PAAD remains a lethal tumour with a high mortality rate, and limited improvement in survival has been achieved in recent years. To gain further insights into the molecular mechanism underlying pancreatic tumorigenesis, publicly available sequencing data were first analysed using bioinformatics methods. After comprehensive analysis of the gene microarray GSE62165, 10 hub genes, including COL10A1, were identified. Furthermore, COL10A1 expression was found to be elevated in pancreatic cancer tissues and cell lines (vs. corresponding controls) and to be inversely related to the prognosis of PAAD patients. The proliferation, invasion, and migration of pancreatic cancer cells were suppressed after COL10A1 knockdown in vitro. In addition, the differentially expressed genes related to COL10A1 in PAAD were analysed by LinkedOmics. CD276 was found to be a target of COL10A1, and knockdown of COL10A1 repressed CD276 expression. Furthermore, COL10A1 promoted PAAD progression by regulating CD276.

The excessive deposition of extracellular matrix (ECM) promotes PAAD tumorigenesis and tumour progression [20]. Among the components of the ECM, collagen is the most abundant. Recent studies have revealed that collagen greatly contributes to the progression of cancers, including pancreatic cancer. According to Duan and his colleagues, type I collagen promotes EMT by regulating the Hedgehog pathway and β1-integrin in pancreatic cancer [21]. Collagen signalling increased tumour progression in a murine model of pancreatic ductal adenocarcinoma after treatment with anti-VEGF therapy [22]. COL10A1 belongs to the collagen family. There is evidence that COL10A1 is overexpressed in a variety of solid tumour types [9, 10]. According to Huang et al., the prognosis of colorectal cancer patients with high COL10A1 expression is worse than that of those with low expression [23]. Moreover, the expression level of COL10A1 in colorectal cancer predicts metastatic and immunogenic properties [24]. COL10A1 promotes the invasion and metastasis of gastric cancer cells, and COL10A1 is regulated by the TGF-β1-SOX9 axis [12]. The results of our study revealed that COL10A1 expression is abnormally elevated in PAAD and related to poor patient outcomes. These results suggest that COL10A1 is a potential prognostic biomarker and therapeutic target in PAAD.

In this study, we identified CD276 as a downstream target of COL10A1. CD276 is a member of the B7/CD28 family and exerts immunosuppressive effects by inhibiting NK cell activity and T-cell proliferation. Cancer stem cells (CSCs) evade immune surveillance by utilizing CD276 in the malignant progression of neck squamous cell carcinoma [25]. CD276 contributes to avoiding cancer cells from immune destruction by shaping the suppressive immune microenvironment [26]. In addition, recent studies have demonstrated that proliferation, migration, invasion, and transition from endothelial to mesenchymal (EMT) are facilitated by CD276 in cancer cells. In pancreatic cancer, CD276 expression was high, and patients with high CD276 expression had a poor prognosis. Both CD276 and PD-1 belong to the B7 family. Immunotherapy with PD-1/PD-L1 inhibitors has been applied for cancer treatment and has shown promising curative effects in some kinds of tumours. However, pancreatic adenocarcinoma is a kind of nonimmunogenic tumour and is resistant to PD-1/PD-L1 inhibitors. The combination of CD276 inhibitors and PD-1/PD-L1 inhibitors in tumour treatment has become a hot research topic. Recently, a study revealed that CD276-targeted photodynamic therapy combined with PD-L1/PD-1 axis inhibition generated local and systemic antitumour responses not only against primary tumours but also against disseminated metastases [27]. PD-L1 and CD276 blockade enhanced the antitumour response in non-small cell lung cancers expressing CD276 [19]. We found that COL10A1 regulates CD276 in this research, which may provide novel insight for future studies investigating immunotherapy in PAAD.

The current study has several limitations. First, we only analysed the gene sequencing data of GSE62165. The combined analysis of other large-scale pancreatic cancer gene expression datasets may be better. Second, we found that COL10A1 was overexpressed in pancreatic cancer tissues and cell lines and associated with patient prognosis. The role of COL10A1 in pancreatic cancer cell proliferation, migration, and invasion was investigated in vitro. Nevertheless, analysis of the biological function of COL10A1 in PAAD in vivo is required in further studies. Third, the regulation of CD276 by COL10A1 should be confirmed by further studies.

In conclusion, using microarray analysis and integrated bioinformatic analysis, the present study identified hub genes involved in PAAD tumorigenesis. COL10A1 was identified as a potential candidate oncogene in PAAD. Furthermore, the role and mechanisms of COL10A1 in PAAD were explored. The results of our research indicated that COL10A1 promotes tumorigenesis by modulating CD276 in pancreatic adenocarcinoma. COL10A1 may be a novel diagnostic or therapeutic target in pancreatic adenocarcinoma.

### Electronic supplementary material

Below is the link to the electronic supplementary material.

**Supplementary Material 1: Figure S1** Panc-1 CD276

**Supplementary Material 2: Figure S2** Panc-1 β-actin. Marker, Panc-1, Panc-1-NC, Panc-1-COL10A1-si-a, Marker, Panc-1, Panc-1-NC, Panc-1-COL10A1-si-a, Marker, Panc-1, Panc-1-NC, Panc-1-COL10A1-si-a, Marker, Panc-1, Marker

**Supplementary Material 3: Figure S3** BXPC-3 CD276

**Supplementary Material 4: Figure S4** BXPC-3 GAPDH. Marker, BXPC-3, BXPC-3-NC, BXPC-3-COL10A1-si-a, BXPC-3-COL10A1-si-a

## Data Availability

The dataset GSE62165 is available in the GEO database (http://www.ncbi.nlm.nih.gov/geo). On the basis of TCGA and GTEx data, GEPIA (http://gepia.cancer-pku.cn/) was applied to analyse the gene expression of COL10A1 in pancreatic adenocarcinoma (PAAD) and normal pancreatic tissues. Based on TCGA data, survival analyses of COL10A1 in PAAD were conducted using the LinkedOmics database (http://www.linkedomics.org/login.php).
