# Supplementary figures and images for: COL10A1 promotes tumorigenesis by modulating CD276 in pancreatic adenocarcinoma
Source: BMC Gastroenterol. 2023 Nov 16;23:397. doi: 10.1186/s12876-023-03045-2 (PMC10652574; doi:10.1186/s12876-023-03045-2)

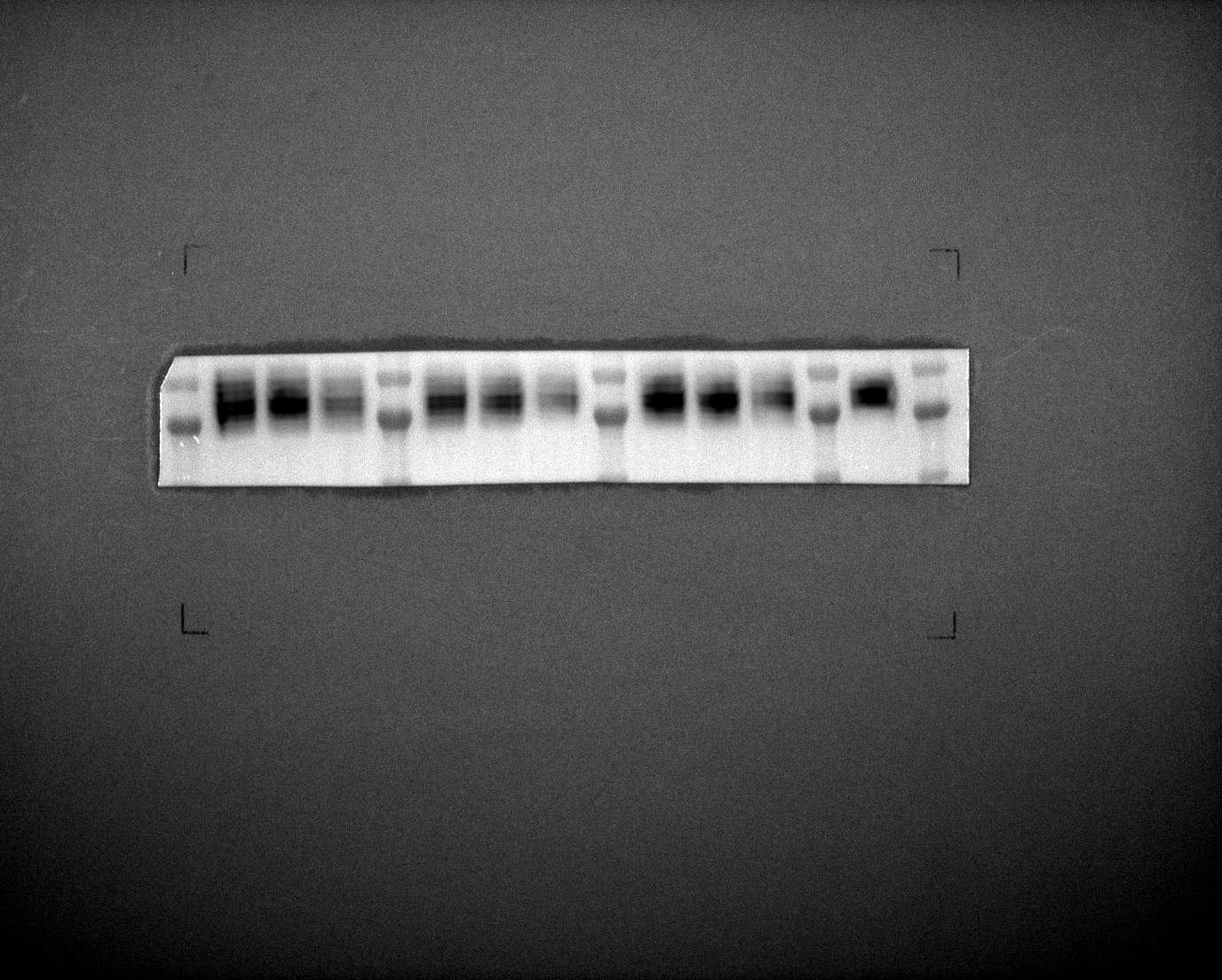

Supplement: Supplementary file 1 — Supplementary Material 1: Figure S1 Panc-1 CD276 [file 12876_2023_3045_MOESM1_ESM.png]

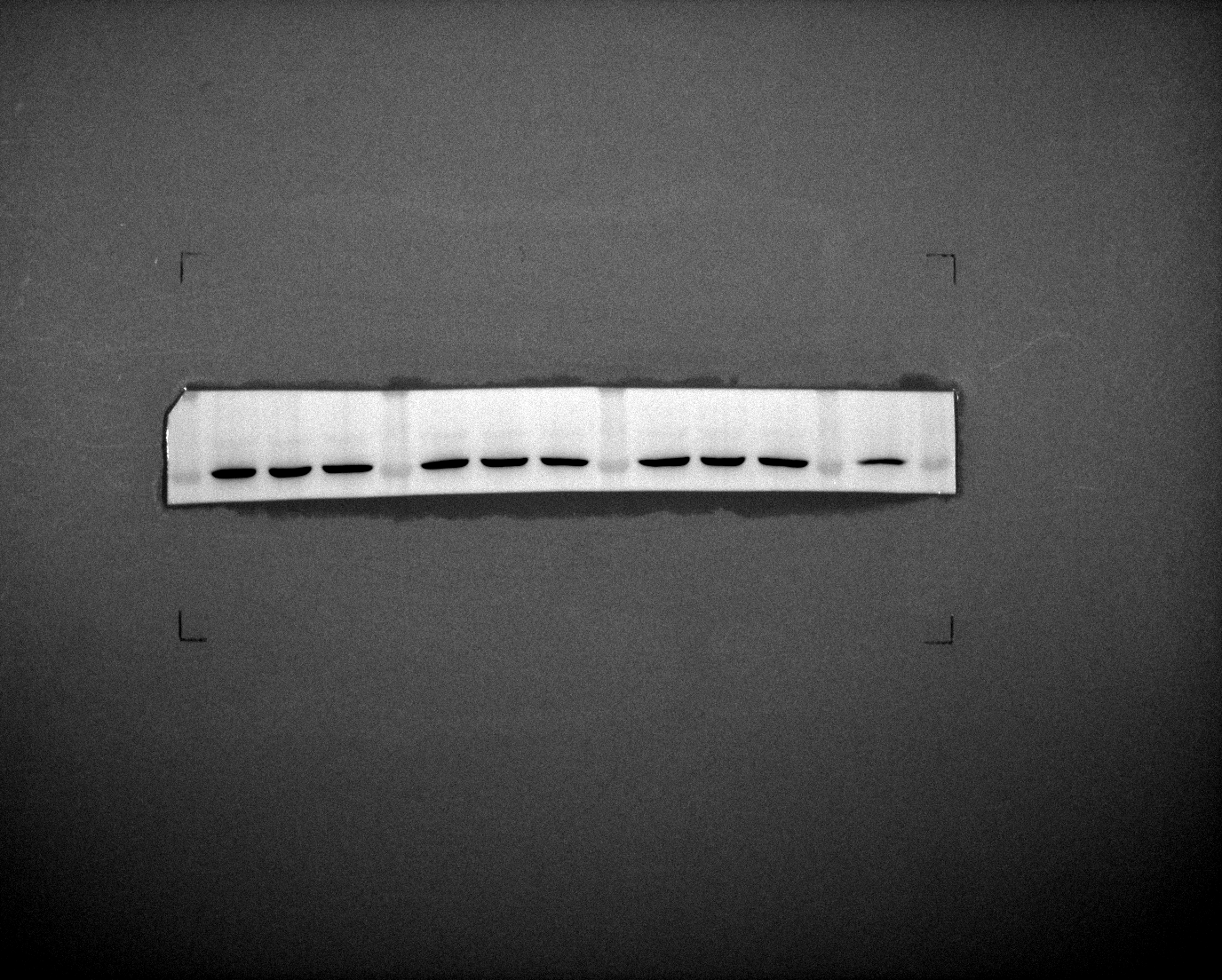

Supplement: Supplementary file 2 — Supplementary Material 2: Figure S2 Panc-1 β-actin. Marker, Panc-1, Panc-1-NC, Panc-1-COL10A1-si-a, Marker, Panc-1, Panc-1-NC, Panc-1-COL10A1-si-a, Marker, Panc-1, Panc-1-NC, Panc-1-COL10A1-si-a, Marker, Panc-1, Marker [file 12876_2023_3045_MOESM2_ESM.png]

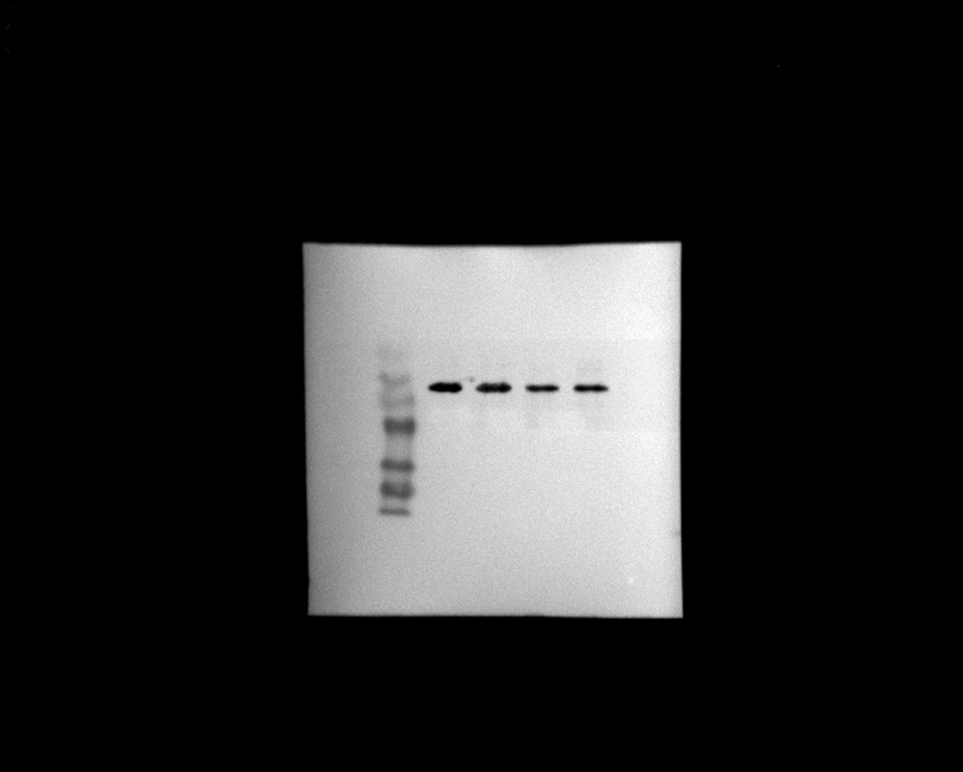

Supplement: Supplementary file 3 — Supplementary Material 3: Figure S3 BXPC-3 CD276 [file 12876_2023_3045_MOESM3_ESM.png]

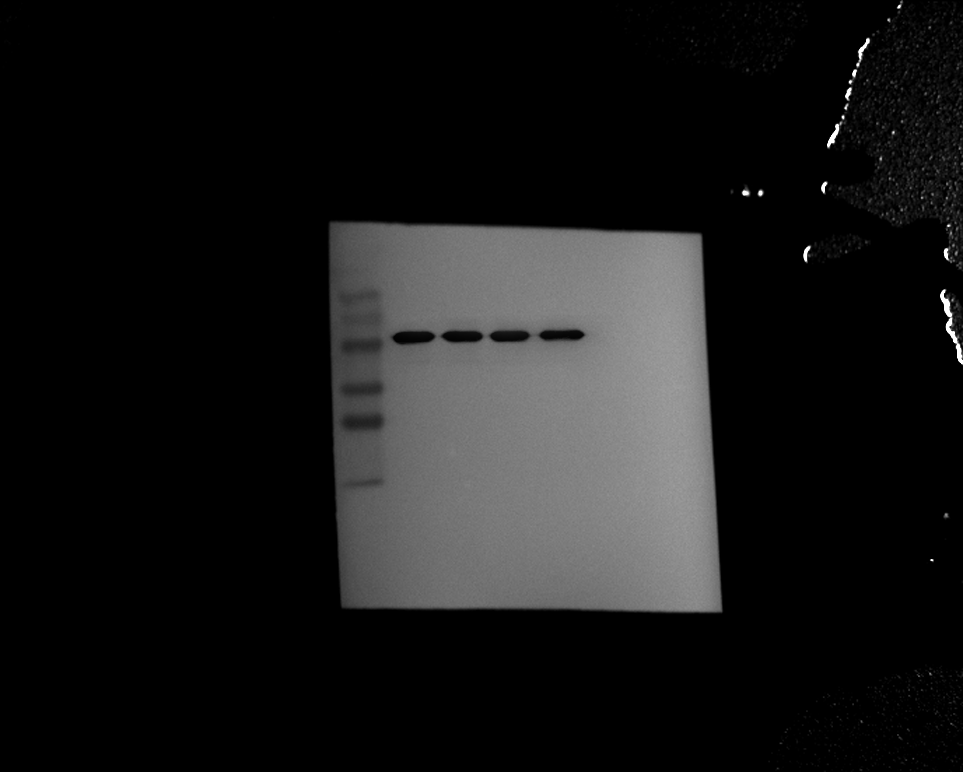

Supplement: Supplementary file 4 — Supplementary Material 4: Figure S4 BXPC-3 GAPDH. Marker, BXPC-3, BXPC-3-NC, BXPC-3-COL10A1-si-a, BXPC-3-COL10A1-si-a [file 12876_2023_3045_MOESM4_ESM.png]
